# Prevalence of *Helicobacter pylori* Infection in a Group of Morbidly Obese Saudi Patients undergoing Bariatric Surgery: A Preliminary Report

**DOI:** 10.4103/1319-3767.70610

**Published:** 2010-10

**Authors:** Ahmad M. Al-Akwaa

**Affiliations:** Department of Medicine, King Abdulaziz Hospital, National Guard Health Affairs, Saudi Arabia

**Keywords:** Bariatric surgery, *Helicobacter pylori*, morbid Saudi obese patients, prevalence

## Abstract

**Background/Aim:**

Earlier reports from Saudi Arabia have shown high prevalence of *Helicobacter pylori* infection. However, recent studies have documented a reduction in the infection prevalence. No prior study has assessed the prevalence in morbidly obese Saudi patients. We aimed to study the prevalence of *H. pylori* infection in a group morbidly obese Saudi patients referred for endoscopy prior to bariatric surgery.

**Materials and Methods:**

We retrospectively reviewed the medical records of all patients who were referred for upper endoscopy prior to bariatric surgery from June 2006 to September 2008. All data were recorded including patient’s demographics, comorbid conditions, endoscopic and histological findings.

**Results:**

There were 62 patients included, 20 males and 42 females. The mean age was 34 years (range 18 – 51) with a mean BMI of 55 Kg/m^2^ (range 35 -92). *H. pylori* were present in 53 patients (85.5%) with chronic active gastritis. All patients with positive *H. pylori* had chronic gastritis of variable severity. Intestinal metaplasia was present in 5%. The prevalence of *H. pylori* infection was similar in patients with and without co-morbid conditions. Main endoscopic findings were gastritis in 67.7%, hiatus hernia in 13%, and gastric erosions in 13%. No patient had duodenal or gastric ulcer.

**Conclusions:**

There is a high prevalence of *H. pylori* infection in morbidly obese Saudi patients undergoing bariatric surgery being referred for upper GI endoscopy. Further prospective studies are needed to evaluate the clinical implication and benefit of eradication treatment of infection in these patients.

*Helicobacter pylori* (HP) infection has been reported to be hyperendemic in Saudi Arabia.[1–5] Reports in the 1990s have shown a prevalence of 68 – 82.2%[1–5] in various age groups of patients including those with non-ulcer dyspepsia. This high prevalence has been attributed to several factors including sanitation and socioeconomic status.

Reports in the 2000s have shown marked reduction in the prevalence to 35 – 55%.[6–8]

In Saudi Arabia, neither the prevalence of (HP) nor the clinical implication of such infection has been looked at in morbidly obese patients undergoing bariatric surgery. Few studies worldwide have shown a high prevalence of HP in morbidly obese patients undergoing bariatric surgery.[9–14]

Our aim in this study was to find out the prevalence of *Helicobacter pylori* infection in a group of morbidly obese Saudi patients referred for elective esophagogastroduodenoscopy (EGD) prior to bariatric surgery.

## MATERIALS AND METHODS

We reviewed the medical records of the patients who were referred for EGD from June 2006 to Sep 2008 before bariatric surgery. Patients were included regardless of the type of surgery they underwent including gastric bypass, biliopancreatic diversion or vertical banded gastroplasty. The main indication for endoscopy was preoperative evaluation. Almost all patients were asymptomatic. Data recorded were patient demographics, co-morbid conditions, medication taken endoscopic and histological findings. All patients had three endoscopic gastric biopsy specimens for the identification of the HP infection, two from antrum and one from the body.

The presence or absence of HP was based on the histological identification of the organism by routine hematoxylin and eosin stain or special methylene blue stains.

## RESULTS

**Figure 1:**
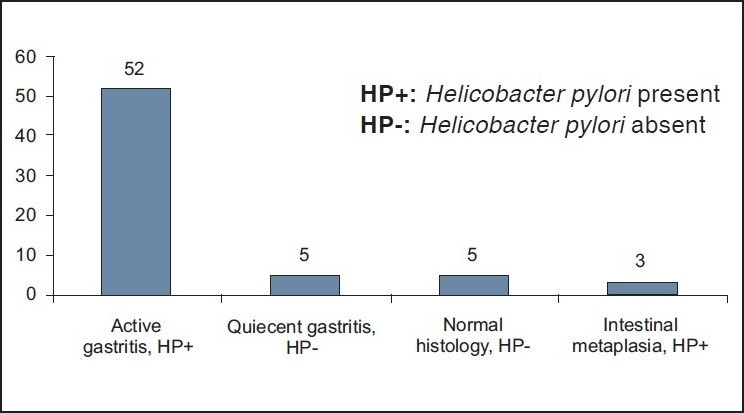
Histological findings in 62 patients

**Figure 2:**
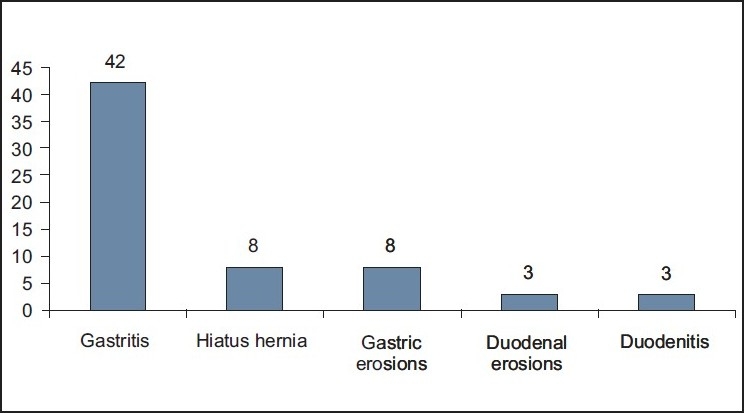
Endoscopic findings in 62 patients highlight this portion

Sixty two patients were referred for upper GI endoscopy as pre bariatric surgery evaluation. Only six patients had mild dyspepsia. The mean age was 34 years (range 18 – 51) and a mean BMI was 55 kg/m^2^ (range 35 - 92). The majority were females: 42 versus 20 males Table 1. *Helicobacter pylori* infection was detected histologically in 53 patients (85.5%). All patients with positive HP organism in their biopsy specimens had chronic active gastritis of variable severe mild, moderate, or severe. Five patients had normal histological findings [Figure 1]. Four patients had quiescent gastritis without evidence of HP organisms. Three patients had intestinal metaplasia in association with HP organisms. *Helicobacter pylori* infection was present in 84% and 83% in patients with and without comorbid conditions, respectively. The main endoscopic findings were gastritis in 42 (67.7%), hiatus hernia in 8 (13%), and gastric erosions in 8 patients (13%) [Figure 2]. No endoscopic evidence of gastric or duodenal ulcer was found.

**Table 1:** Demographic data and co-morbid conditions in 62 patients who underwent bariatric surgery

Sex [male/female]	20/42
Age [years]*	34 (18 – 51)
Weight [Kg]*	145 (69 – 280)
Body mass index [kg/m^2^]*	55 (35 – 92)
Diabetes mellitus	28
Hypertension	22
Bronchial asthma	6
Hypothyroidism	3
Obstructive sleep apnea	3

* Mean (range)

## DISCUSSION

A high prevalence of HP infection in Saudi Arabia has been shown in various earlier studies.[1–5] A trend toward increasing prevalence with advancing age has also been shown[2,3,5,7] and a higher prevalence of 93% was reported in patients with duodenal ulcers.[3]

Significant reduction in infection rate has been demonstrated in recent reports[6–8] and a reduction of prevalence down to 63% has been reported in the population with duodenal ulcers.[15]

A prevalence rate as low as 35% has been reported in a random sample of medical students.[6]

No information in the literature is available to show the prevalence of HP infection in patients undergoing bariatric surgery from this country or nearby countries.

This study has shown an exceptionally high prevalence of the infection in unselected group of morbidly obese patients in a relatively young age group who have undergone EGD before weight reduction surgeries.

The explanation of such a high prevalence is not clear and has not been clarified.

It could probably be related to the study design since it was a retrospective analysis, although this effect would have been offset by a representative sample where the patients inclusion was random.

The basis of referring the patients for EGD was preoperative screening which was thought to be necessary by some surgeons and not by others. The difference in surgeons’ views was the reason for referring some patients for screening EGD while not for the others.

Several reports from the west have shown a prevalence of 11 – 39% in the morbidly obese group who underwent bariatric surgery; and the rates were higher than similar non-obese groups [Table 2].[9–13] One recent study from USA has found a higher prevalence rate of 61% in morbidly obese compared to 48% in the control group, but this study looked only at the *H. pylori* serologies, which might not reflect a true active infection [Table 2].[14]

**Table 2:** Studies on the prevalence of HP in morbidly obese patients who underwent bariatric surgery

Authors, year/type of study	No. of patients	HP prevalence (%)	Control	HP (%)
Renshaw *et al*.[9] 2001/ Retrospective	56	38	500	21
Papasavas *et al*.[11] 2008/ Retrospective	259	22.4		
Vanek *et al*.[12] 2006/ Retrospective	96	11		
De moura *et al*.[13] 2008/ Prospective	96	37.5		
Erim *et al*.[14] 2008/ Prospective	240	61		48
Azaqury *et al*.[16] 2006/ Retrospective	319	39		
Schermier *et al*[18] 2002/ Prospective	206	30		
Current study retrospective	62	85.5		

HP: *Helicobacter pylori*

Certain implications of the HP infection in bariatric surgery patients have been shown by few studies. These clinical implications include postoperative foregut symptoms, postoperative marginal ulcers, amount of weight loss, and delay of surgery.[10,16–18] In order to avoid these problems, eradication of *H. pylori* has been warranted after confirming the diagnosis.

Intestinal metaplasia is considered a premalignant condition, which is related to *Helicobacter pylori* infection. Around 5% of our patients had intestinal metaplasia. It necessitates treatment with eradication therapy, followed by endoscopic and histological examinations. The metaplasia in our group is lower than reported previously[19] but has clinical importance from the management point of view.

Unlike previous reports, this study has not shown a difference in rate of HP infection between patients with and without comorbid conditions.[20–22]

It might be necessary to screen these patients for the HP infection and give eradication treatment before subjecting them for surgeries.

We may need to follow our patients and expand the study prospectively and assess the prevalence of the infection in larger group of patients and observe the consequences postoperatively and examine the benefit of eradication treatment.

The study, nevertheless, has some limitations. It represents a retrospective chart review with a small sample size. However, it is the only preliminary report from Saudi Arabia and the results could reflect the persistence of the high prevalence of HP in a relatively young age group which probably could indicate hyperendemicty of the infection in Saudi Arabia.

## CONCLUSIONS

The study demonstrated a very high prevalence of *Helicobacter pylori* infection in morbidly obese Saudi patients who underwent bariatric surgery.

We may need to do more prospective studies to examine the implication of the infection and importance of HP eradication in this group of patients.
